# Polyethylene liner dissociation in total hip arthroplasty: a retrospective case–control study on a single implant design

**DOI:** 10.1186/s10195-024-00785-z

**Published:** 2024-08-14

**Authors:** S. Pagano, J. F. Plate, T. Kappenschneider, J. Reinhard, M. Scharf, G. Maderbacher

**Affiliations:** 1 Department of Orthopaedic Surgery, University of Regensburg, Asklepios Klinikum, Kaiser-Karl-V.-Allee 3, 93077 Bad Abbach, Germany; 2 Department of Orthopaedic Surgery, University of Pittsburgh Medical Center, Pittsburgh, PA USA

**Keywords:** Total hip arthroplasty, Liner dislocation, Polyethylene, Modularity, Component positioning, Case–control study

## Abstract

**Background:**

Modular acetabular components for total hip arthroplasty (THA) provide intraoperative flexibility; however, polyethylene liner dissociation may occur. This study aimed to examine the incidence and causes of liner dissociation associated with a specific acetabular component design at a single centre.

**Materials and methods:**

A retrospective analysis of 7027 patients who underwent primary THA was performed to identify isolated liner dislocations. Patient demographics, clinical presentations, surgical and implant details, and both radiographic and computed tomography (CT) findings were analysed. Patients with liner dislocation were matched to a control group via 2:1 propensity score matching, and a logistic regression analysis was employed to identify associated risk factors.

**Results:**

A total of 32 patients (0.45%) experienced liner dislocation at a mean 71.47 ± 60.10 months post surgery. Significant factors contributing to dislocations included the use of a conventional compared with a highly crosslinked polyethylene component (*p* = 0.049) and screw fixation (*p* = 0.028). Radiographic and CT analysis highlighted the importance of proper component orientation, revealing that patients experiencing dislocations demonstrated significantly lower acetabular cup anteversion angles (*p* = 0.001) compared with the control group. Impingement and malposition, identified in 41% and 47% of the cases, respectively, further underscored the multifactorial nature of dislocation risks.

**Conclusions:**

While the overall rate of polyethylene liner dislocation was low, the findings of this study highlight the importance of appropriate cup placement to decrease the risk of dissociation. It further substantiates the influence of impingement and malposition in liner displacement, with increased mechanical stress exerted on the locking mechanism under adverse conditions and the potential risk increase due to screw placement.

## Introduction

Total hip arthroplasty (THA) is one of the most common surgical interventions worldwide [1]. As the global population ages, projections suggest that, by 2050, an even larger segment will require this surgical intervention, underscoring its essential role in preserving mobility and enhancing quality of life [2]. Modularity in THA offers significant intraoperative flexibility, allowing surgeons to adjust the length, offset, and anteversion of the components. Yet, while this modularity enhances versatility, it can also introduce potential failure modes. Specifically, wear on the backside and disassociation of the polyethylene liner from the acetabular shell have been observed [3]. Several manufacturers with modular designs, including Pinnacle^®^ (DePuy, Johnson & Johnson), Harris-Galante^®^ (Zimmer), Trident^®^ (Stryker), and G7^®^ cup (Zimmer-Biomet), have encountered issues related to liner dissociation [3, 4]. Despite the 10-year survival rate of up to 99.2% using the Pinnacle acetabular system [5–7], there are several reported instances of cup/liner dissociation [8–14].

The incidence of cup/liner dissociation in this system is reported between 0.17% and 2.40%, requiring revision surgery associated with increased resource utilization and morbidity to the patient that make this entity a matter of pressing concern [8, 9]. Different liner options, such as neutral, lipped, lateralized, and the 10° face changing variants, have shown differential rates of dissociation. Notably, neutral and face-changing liners appear to have a propensity for higher dissociation rates compared with their lipped counterparts [15]. However, comprehensive studies focussing on the underlying reasons for the dissociation of the liner remain scant [8]. Given the potential morbidity associated with liner dissociation, which necessitates surgical revision, a more in-depth exploration is necessary.

This retrospective observational study aims to further explore this concern by investigating the frequency and underlying causes of dissociation of the liner in a cohort of patients who have undergone a THA with this acetabular system at a single institution since 2010. The study hypothesized that acetabular component position influences the risk of polyethylene liner dissociation.

## Materials and methods

### Study design and setting

**Fig. 1:**
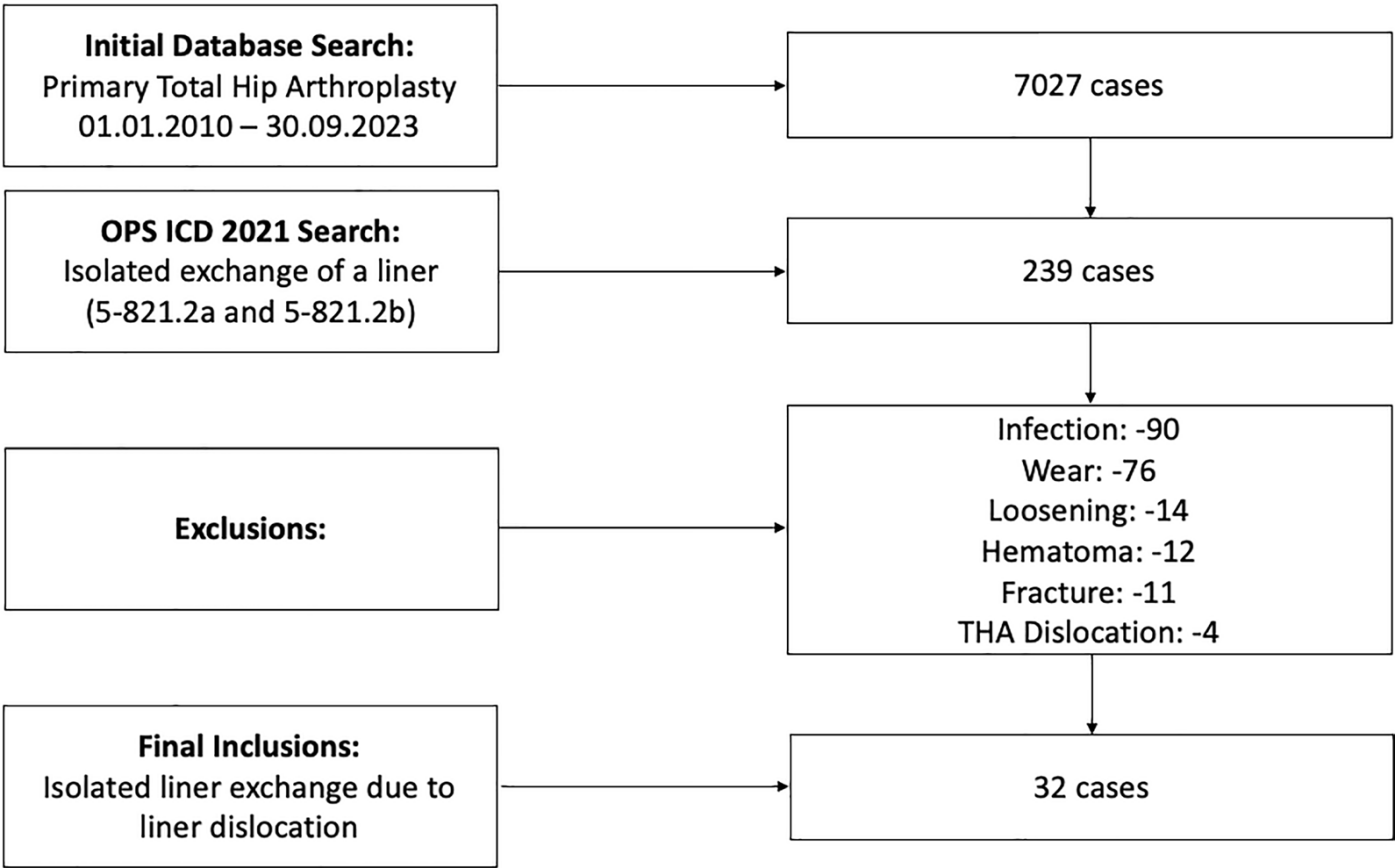
Flowchart illustrating the database search process divided into two distinct categories: primary total hip arthroplasty (THA) and THA revision refined using specific OPS codes for isolated exchange of the liner

A retrospective observational study was conducted at a single institution to assess the incidence and underlying causes of Pinnacle (DePuy, Johnson & Johnson) cup/liner dissociation. Between 2010 and 2023, the period covered by our electronic records, a total of 7027 primary THA procedures were identified in which this acetabular system was implanted. Using the OPS ICD 2021 codes 5–821.2a and 5–821.2b, a total of 239 THA liner exchanges were identified and further analysed. From this group, 207 cases were excluded after reviewing the ICD-10-GM codes that documented the reasons for liner exchange. The excluded cases involved liner exchange due to infections, component loosening, hematomas, hip dislocations without evidence of liner dissociation, and liner wear. Only patients who had undergone liner exchange specifically due to dissociation of the polyethylene liner, confirmed by image analysis and operative note review, were included in the final analysis. After this selection process, the final cohort consisted of 32 patients suitable for analysis and a comprehensive set of data was collected from the internal clinical database (ORBIS, Agfa healthcare) including patient demographics, surgical variables, and clinical findings (Fig. 1).

The suspected cause of the dislocation, and the period between symptom onset and the surgical intervention were documented. The type of primary implantation, the surgical approach employed, head and neck lengths, specifics about the dislocated liner, the age of the implant at the time of liner exchange, intraoperative findings, and other crucial details about the implant components were also assessed.

### Measurements

**Fig. 2:**
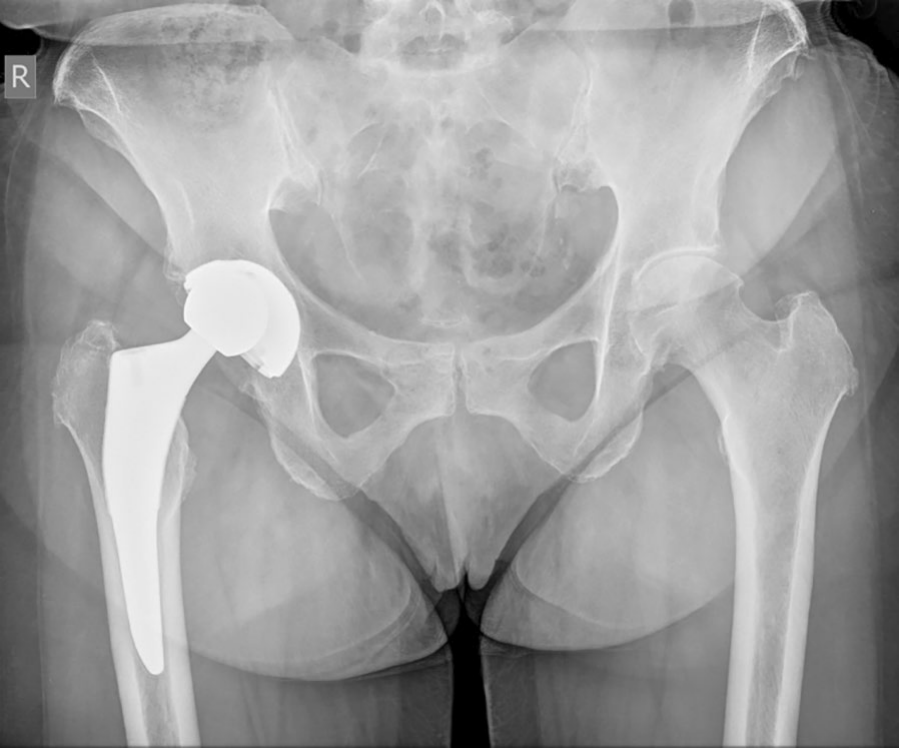
Anteroposterior X-ray of the pelvis showing a dislocation of the femoral head caused by liner dissociation from the acetabular cup. The image clearly depicts the contact of the dislocated femoral head with the acetabular cup

Anteroposterior pelvic radiographs were retrieved from the last postoperative follow-up examination before the dislocation of the liner and, when available, from extremity rotational CT scans, taken at the time of dislocation. X-ray evaluations were not conducted on the radiographs obtained at the time of dislocation, as the displaced head would have influenced the leg length discrepancy and obscured the acetabular cup rim (Fig. 2). The measurements were obtained using radiographs with a graduated sphere of 25 mm and processed via Medicad software (Hemtec, Landshut, Germany).

**Fig. 3:**
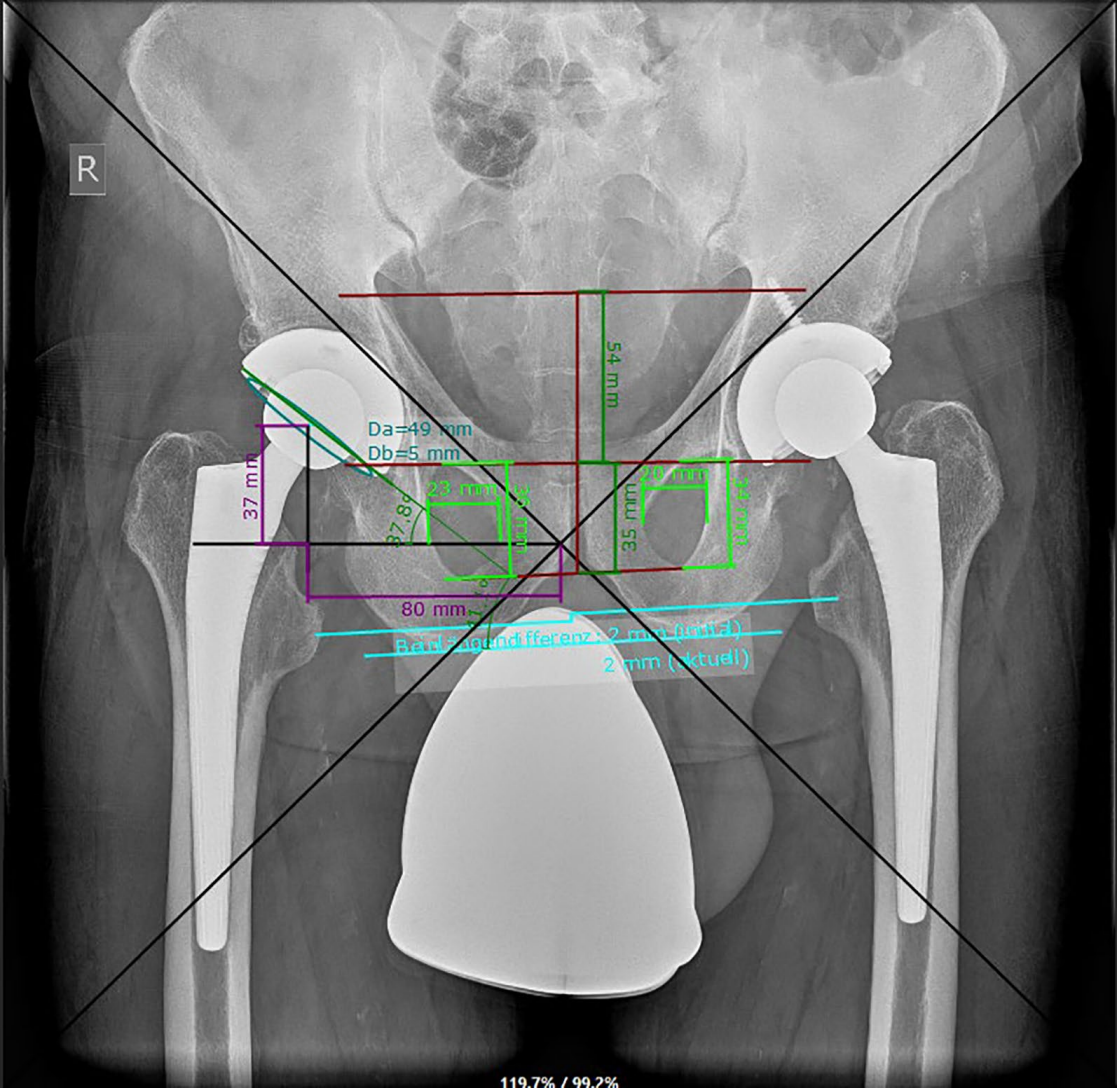
The acquired values were input into an algorithm that calculated the acetabular cup’s anteversion and inclination on the anterior pelvis plane (APP) and the vertical functional coronary plane (FCP)

To evaluate the acetabular anteversion and inclination on both the anterior pelvis plane (APP) and the vertical functional coronary plane (FCP) (Fig. 3), we utilized an algorithmic model developed by Schwarz et al. [16, 17]. This model helps accurately assess the orientation of the acetabular cup on pelvic radiographs by correcting for potential errors caused by the positioning of the X-ray beam. Specifically, it adjusts for deviations in the central beam that can lead to inaccurate measurements of cup anteversion and inclination. The algorithm takes into account the effects of pelvic tilt and rotation, which can distort the appearance of the cup on radiographs. By applying mathematical corrections, it compensates for these distortions, allowing for a more accurate assessment of the cup’s orientation [16, 17]. Experimental results using a dummy pelvis demonstrated that the corrected measurements had an average absolute difference of only 0.4° for anteversion and 0.3° for inclination, compared with other methods that showed much larger errors [16]. Additionally, the algorithm was tested with various pelvic positions, confirming its robustness across different scenarios [17].

Furthermore, an algorithm by Kanazawa et al. was employed to determine the rotation of the pelvis [18]. This algorithm quantifies three-dimensional (3-D) pelvic rotation using the width and height ratio of the obturator foramina under various pelvic tilts, thus enabling an estimation of pelvic rotation on plain anteroposterior radiographs. The algorithm’s validity was confirmed by comparing the calculated rotations with known values on reconstructed pelvises from CT scans. The authors demonstrated a strong linear correlation between the width-to-height ratio (WR) of the obturator foramina and axial pelvic rotation, with correlation coefficients ranging from 0.94 to 0.98, regardless of sagittal pelvic tilt up to 10° anteriorly [18].

**Fig. 4:**
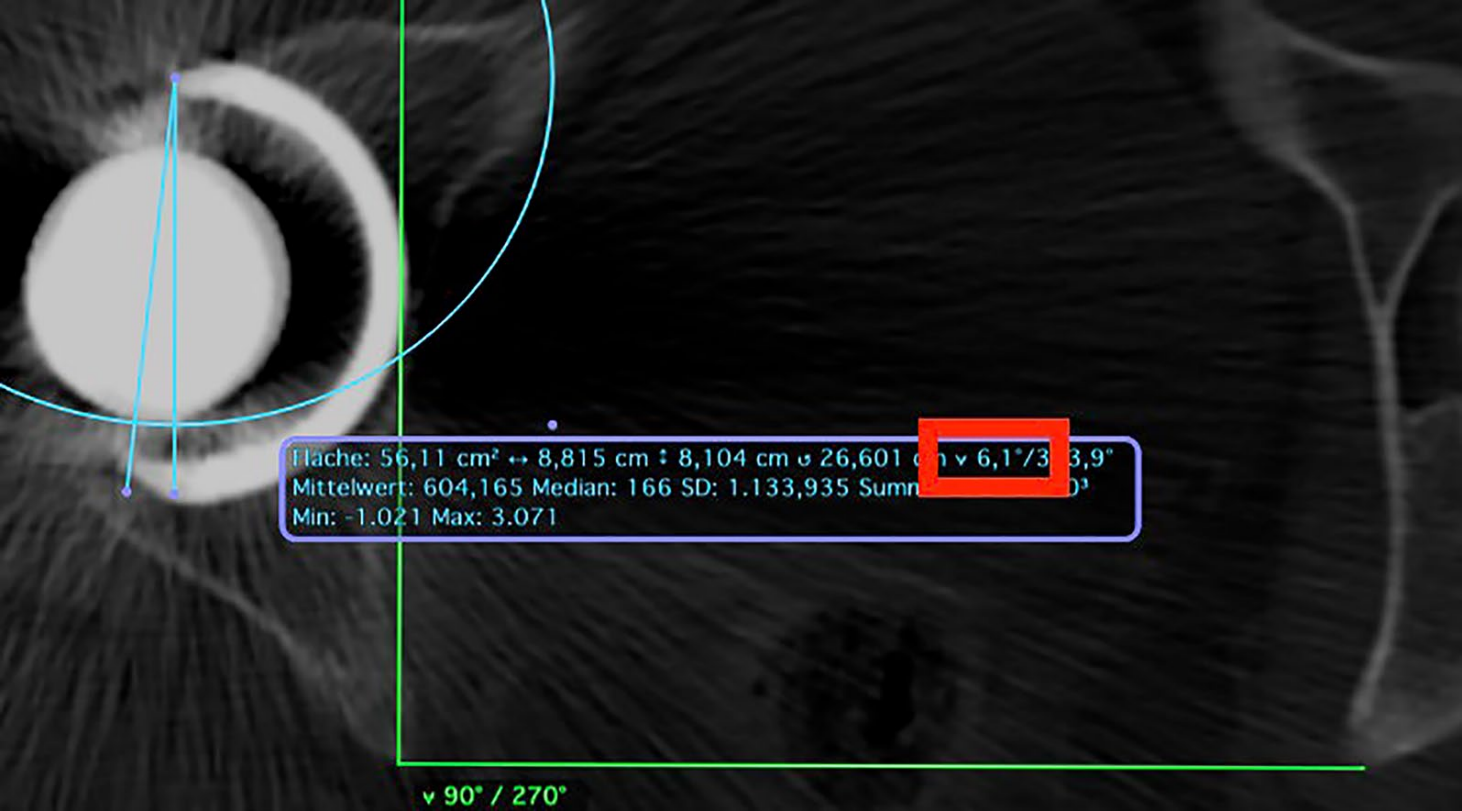
Rotational CT scans of the lower extremities were assessed using the software OsirixLite (Pixmeo, Bernex, Switzerland). In the provided example, the cup’s anteversion is measured

For the rotational CT scans of the lower extremities, we utilized OsirixLite software (Pixmeo, Bernex, Switzerland) to assess the images. In the CT scans, we determined the acetabular cup anteversion on the APP, the stem anteversion, and the combined anteversion. To measure the CT scans, we adopted the prevalent method in the current literature [19, 20]. The cup’s anteversion was determined using the angle between a line that connects the cup’s lateral anterior and posterior edges and a perpendicular line linking two symmetrical points on each side of the pelvis. The stem’s anteversion, which reflects the relationship between the condylar axis and the stem neck, was measured using a line drawn between the posterior portions of the medial and lateral femoral condyles and another line that joins the centre of the femoral head to the femoral component’s neck centre. The combined anteversion was obtained by summing the angle of the cup and the femoral component’s anteversion (Fig. 4). All measurements were conducted by a single senior surgical resident (S.P.) and subsequently assessed, verified, and confirmed by a senior surgeon (G.M.).

### Statistical analysis

Continuous variables are described using mean values accompanied by their standard deviation (SD). Categorical data are presented as absolute counts (*n*) and associated percentages (%).

A matched control group was established via a 2:1 propensity score matching method, implemented using XLSTAT (Lumivero, Denver, USA). The 32 patients with cup/liner dissociation were aligned with 64 corresponding controls from the original pool of 7027 hip arthroplasty cases. The matching was based on age and body mass index (BMI). We employed the Mahalanobis distance within a calliper of 0.2 standard deviations of the propensity score’s logit. This statistical method was used to control for confounding variables, ensuring that the matched groups were comparable and reducing the risk of confounding bias.

Continuous variables were analysed using independent *t*-tests, while categorical variables were examined using chi-square tests. Logistic regression was performed to identify independent risk factors significantly associated with liner dislocation. All statistical tests were two-sided, with a significance level set at *p* < 0.05.

IBM SPSS Statistics (IBM Corp, New York, USA) version 29 was employed for the statistical analyses. The study adhered to institutional ethical standards and the Declaration of Helsinki (protocol no. 23-3384-104, 19 May 2023).

## Results

### Demographics and clinical presentation

Of the 7027 patients who underwent primary THA during the study period, 32 experienced dislocation of the liner, leading to a 0.45% complication rate. The average age at the time of revision surgery for polyethylene liner exchange was approximately 72 years (SD 9.95 years). All patients with a dislocated liner reported a squeaking noise from their hip, and in half (50.0%), no discernible cause for the dislocation was identified. Detailed demographics, clinical presentations, and causes can be found in Table 1.

**Table 1:** Summary of demographics, symptoms, and causes for Pinnacle liner dissociation in the patient court

Demographics	Dislocated liners (*n* = 32)
Age (y)	
Range	53–88
Mean (± SD)	71.66 (± 9.95)
BMI (kg/m^2^)	
Range	20.20–44.01
Mean (± SD)	29.5 (± 4.96)
Sex	
Women	13 (40.6%)
Men	19 (59.4%)
Pain	
Yes	19 (59.4%)
No	13 (40.6%)
Squeaking noises	
Yes	32 (100%)
No	0 (0%)
Duration of symptoms (days)	
Range	1–84
Mean (± SD)	18.72 (± 25.77)
Cause of dislocation	
Squatting	4 (12.5%)
Bucking	5 (15.6%)
Lifting	2 (6.3%)
Stumbling	4 (12.5%)
Leg external rotation	1 (3.1%)
None apparent cause	16 (50.0%)

### Surgical and implant data

A significant association between the type of liner used and the risk of dislocation was found. Patients with conventional polyethylene (Marathon) were approximately 2.8 times more likely to experience dislocation compared with those with highly crosslinked polyethylene (Altrx) (odds ratio [OR] = 2.797, 95% confidence interval [CI] = 1.002 to 7.807, *p* = 0.049). The employment of fixation screws was also found to be associated with an increased risk of liner dislocations (OR = 11.667, 95% CI = 1.301 to 104.647, *p* = 0.028). Surgical details and component specifics are outlined in Table 2.

**Table 2:** Surgical details (a) and implant specifics (b) for patients with Pinnacle liner dissociation compared with the control group

	Dislocated liners (*n* = 32)	Control group (*n* = 64)	*p*-Value
(a) Surgical details			
Side			
Right	20 (62.5%)	33 (51.6%)	0.639
Left	12 (37.5%)	31 (48.4%)	
ASA			
1	3 (9.4%)	9 (14.1%)	0.348
2	22 (68.8%)	34 (53.1%)	
3	7 (21.9%)	21 (32.8%)	
Time from THA (m)			
Range	0–203	–	–
Mean (± SD)	68.41 (± 60.13)	–	
Surgical approach			
MI anterolateral (lateral decubitus)	29 (90.6%)	56 (87.5%)	0.652
Lateral (supine position)	3 (9.4%)	8 (12.5%)	
(b) Implant specifics			
Cup diameter (mm)			
48	3 (9.4%)	2 (3.1%)	0.386
50	2 (6.3%)	13 (20.3%)	
52	10 (31.3%)	17 (26.6%)	
54	5 (15.6%)	13 (20.3%)	
56	5 (15.63%)	10 (15.6%)	
58	4 (12.50%)	3 (4.7%)	
60	3 (9.4%)	5 (7.8%)	
62	0 (0.0%)	1 (1.6%)	
Screw fixation			
Yes	5 (15.7%)	1 (1.6%)	**0.028**
No	27 (84.4%)	63 (98.4%)	
Liner model			
Marathon	23 (71.9%)	37 (57.8%)	**0.049**
Altrx	6 (18.8%)	27 (42.2%)	
N/A	3 (9.4%)	–	
Liner diameter (mm)			
28	2 (6.3%)	1 (1.6%)	0.699
32	30 (93.8%)	63 (98.4%)	
4-mm offset liner			
Yes	2 (6.3%)	6 (9.4%)	0.644
No	30 (93.8%)	58 (90.6%)	
10° face changing liner			
Yes	2 (6.3%)	0 (0.0%)	0.999
No	30 (93.8%)	64 (100.0%)	
Head length (mm)			
1	12 (37.5%)	35 (54.7%)	0.054
5	12 (37.5%)	19 (29.7%)	
9	6 (18.8%)	10 (15.6%)	
13	1 (3.1%)	0 (0.0%)	
17	1 (3.1%)	0 (0.0%)	
Head material			
Metal	31 (96.9%)	56 (97.5%)	0.170
Ceramic	1 (3.1%)	8 (12.5%)	
Stem Model			
Corail	19 (59.4%)	42 (65.6%)	0.835
Tri-Lock	9 (28.1%)	22 (34.4%)	
Link	1 (3.1%)	0 (0.0%)	
Proxima	2 (6.3%)	0 (0.0%)	
Fjord	1 (3.1%)	0 (0.0%)	
High-offset			
Yes	15 (46.9%)	32 (50.0%)	0.773
No	17 (53.1%)	32 (50.0%)	

Statistically significant values (*p* < 0.05) are highlighted in bold

*m* months, *SD* standard deviation, *MI* minimal invasive

N/A: data not available

### Radiographic and CT measurements

Comparison of acetabular cup inclination angles revealed minimal disparity averaging 45.39° ± 6.24° in the dislocated liner cohort versus 44.33° ± 5.85° in the control group, a difference not reaching statistical significance (*p* = 0.418). However, 14 out of 30 acetabular cups (47%) in patients with liner dissociation were positioned outside the Lewinnek safe zones, which are defined as an inclination of 40 ± 10° and an anteversion of 15 ± 10° [21]. In comparison, 25 out of 64 acetabular cups (39%) in the control group were outside these safe zones. This difference was not statistically significant (*p* = 0.486).

Notably, a significant discrepancy was observed in the acetabular cup anteversion angles, as determined using the Lewinnek measurement method. The group with dislocated liners exhibited a mean anteversion angle of 10.38° (± 7.75), markedly lower than the 16.65° (± 8.15) observed in the control group (OR = 0.903, 95% CI = 0.848 to 0.962, *p* = 0.001).

CT measurements, available only in the case group, showed an average cup anteversion of 15.31 ± 14.57° and a combined anteversion of 34.00 ± 16.34°. Two patients’ X-rays and CT rotational analysis for 13 patients with dislocated liner were not available (Table 3).

**Table 3:** X-ray (a) and CT (b) radiographic measurements: ranges, averages, and standard deviations

(a) X-ray measurements	Dislocated liners (*n* = 30)^1^	Control group (*n* = 64)	*p*-Value
Leg length difference (mm)			
Range	−16 to 21	−11 to 18	0.894
Mean (± SD)	1.07 (± 7.52)	0.86 (± 6.91)	
Cup inclination (°)			
Range	31.40 to 60.60	29.40 to 57.90	0.418
Mean (± SD)	45.39 (± 6.24)	44.33 (± 5.85)	
Cup anteversion (°)			
Range	2.29 to 30.95	0.00 to 35.90	**0.001**
Mean (± SD)	10.38 (± 7.75)	16.65 (± 8.15)	
APP cup anteversion (°)			
Range	−12.70 to 50.98	−5.40 to 32.00	0.274
Mean (± SD)	10.69 (± 11.91)	13.26 (± 9.90)	
APP cup inclination (°)			
Range	32.46 to 58.69	27.70 to 59.80	0.308
Mean (± SD)	43.70 (± 6.46)	45.15 (± 6.40)	
FCP cup anteversion (°)			
Range	−3.53 to 48.52	−3.20 to 37.50	0.174
Mean (± SD)	12.68 (± 11.24)	15.66 (± 9.08)	
FCP cup inclination (°)			
Range	33.18 to 58.69	28.50 to 59.20	0.313
Mean (± SD)	44.21 (± 6.36)	45.62 (± 6.26)	

(b) CT measurements	Dislocated liners (*n* = 18)^**2**^
Cup anteversion (°)	
Range	−13.70 to 34.10
Mean (± SD)	15.31 (± 14.57)
Stem anteversion (°)	
Range	−23.20 to 43.20
Mean (± SD)	17.48 (± 18.22)
Combined anteversion (°)	
Range	−8.50 to 60.20
Mean (± SD)	34.00 (± 16.34)

No CTs were available in the control group

^1^X-ray data were not available for two patients

^2^Lower extremity rotational CT was available in 17 cases. For one patient, only a CT scan of the pelvis was available

*SD* standard deviation, *APP* anterior pelvis plane, *FCP* vertical functional coronary plane

### Intraoperative findings

**Fig. 5:**
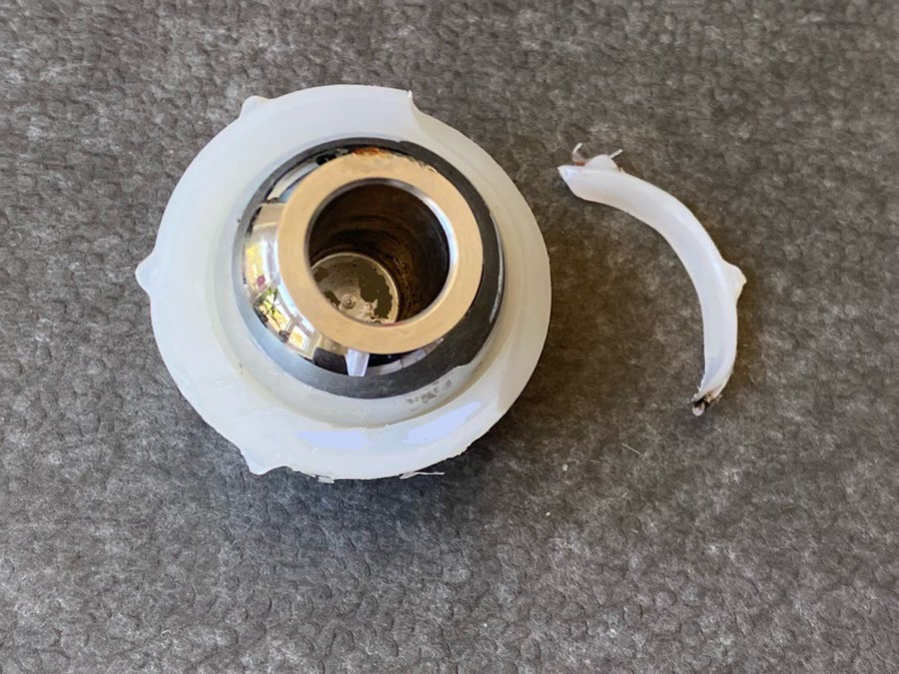
In this particular case the replaced liner shows damage on one edge, with no signs of wear or ovalization. The image has been edited to obscure the serial numbers

During surgery, the most frequent dislocation direction of the liner was caudal, occurring in eight cases (25.0%). Deformations and broken liners were identified in eight (25.0%) and six (18.8%) patients, respectively (Fig. 5). Metallosis was observed in 17 cases (53.1%), and impingement was evident in 13 cases (40.6%) (Table 4).

**Table 4:** Distribution and frequencies of dislocation direction, liner alterations, metallosis, and presence of impingement

Intraoperative findings	Dislocated Liners (*n* = 32)
Dislocation direction	
Ventral	5 (15.6%)
Dorsal	5 (15.6%)
Caudal	8 (25.0%)
N/D	14 (43.8%)
Liner deformation	
Yes	8 (25.00%)
No	2 (6.3%)
N/D	22 (68.8%)
Broken liner	
Yes	6 (18.8%)
No	9 (28.1%)
N/D	17 (53.1%)
Metallosis	
Yes	17 (53.1%)
No	12 (37.5%)
N/D	17 (53.1%)
Impingement	
Yes	13 (40.6%)
No	19 (59.4%)

*N/D* not described

### Revision surgery outcomes

Three patients experienced re-dislocation after their first revision surgery, during which an identical liner was substituted to address the initial dislocation. These patients required a second revision surgery to manage the subsequent dislocation.

Among the patient cohort, four cases during the first revision surgery necessitated not only the replacement of the liner but also the reimplantation of the metal cup due to significant damage. For all other revision surgeries, the procedures generally involved only exchanges of the liner and head components.

## Discussion

The current study underscores that, while the incidence of liner dissociation is low (0.45%), it remains a serious complication in THA patients requiring revision surgery, supporting earlier findings [8–15]. Perkins et al.’s review of literature from 2009 to 2020 found 53 cases of liner dislocations across different studies, with an incidence rate ranging from 0.17% to 2.40%. They also reported 26 instances of dissociation within their own group of 212 patients; all identified cases necessitated surgical revision owing to dissociation [8]. Their analysis suggests that implant malposition, impingement during movement, and potential issues with the locking mechanism of cups are significant factors contributing to dislocation occurrences [8]. Ensuring proper implant positioning and considering potential issues with the locking mechanism during the initial surgery could reduce the incidence of these complications.

However, while malposition alone may not directly lead to liner dislocation, the findings of the current study indicate that acetabular component anteversion may affect dislocation risk, since a higher degree of Lewinnek anteversion was associated with a reduced risk of liner dislocation. This protective mechanism is likely owing to the reduction in impingement that occurs during gait and activities requiring maximal leg flexion, since increased anteversion of the cup has been associated with enhanced range of motion and a reduced risk of impingement, as established by prior studies [22–24]. A lower anteversion angle of the acetabular cup could increase mechanical stress at the extreme range of motion, putting disproportionate stress on the liner against the head and elevating the likelihood of dislocation due to extreme movements acting as leverage. Surgeons should strive for optimal acetabular component positioning, particularly ensuring appropriate anteversion angles to minimize impingement and mechanical stress on the liner.

Beckmann et al. noted that factors such as impingement, malposition (especially during early dissociation), age-related deterioration of the liner, and increased ranges of motion in later stages play vital roles in liner dissociation [25]. These factors often lead to heightened stress and torsion on the locking mechanism, resulting in liner movement and potential dissociation, particularly if coincident damage occurs at the impingement site. However, excessively high anteversion angles can conversely increase wear and deterioration of the polyethylene liner if exceeding 25° [26]. Although recent literature challenges the established “safe zones” proposed by Lewinnek as definitive benchmarks for acetabular implant positioning, they continue to serve as clinical references due to their historical importance and the lack of universally accepted alternatives [26–29].

Perkins et al. [8] noted in their systematic review that 46% out of 53 dissociated liners were lipped, suggesting an increased risk of impingement during normal walking cycles. However, the current study observed a lower frequency of specific liner types, including 4-mm offset and 10° face changing liners, within the current cohort, without a significant correlation to disassociation events. The choice of liner type should consider the potential risk of impingement and dissociation, even though no significant correlation was found in this study, highlighting the complexity of implant selection.

While malposition is often cited as the primary cause of liner dislocation [8], various hypotheses exist concerning the reasons behind the dislocation of modular polyethylene liners [13, 14]. The study by Beckmann et al. emphasized the essential role of anti-rotation tabs (ARTs) in the liner locking mechanism [25]. Their mechanical tests revealed that the absence of ARTs in the Marathon liner resulted in a lower lever-out force required for separation of the cup and liner in this system compared with other tested constructs. This finding aligns with observations in this study, which indicated an increased risk of dislocation associated with the Marathon compared with Altrx liner. Notably, during revision surgeries for disassociations, it was often discovered that the Marathon liners lacked at least three ARTs, a situation that contributes to liner rotation and displacement [11, 12, 14, 15, 25, 30, 31]. However, the lack of detailed records on the integrity of the liner’s ARTs in the current study’s documentation limits further analysis.

The potential influence of implant design features, such as the prominence of screw heads, on liner dissociation has been also previously noted [9]. Although this risk is inherent in modular designs, this acetabular cup appears to exhibit a disproportionately higher rate of this complication.

The findings of this study confirm that screw fixation represents a risk factor for liner disassociation, and avoiding it where possible could help reduce the incidence of this complication.

The current study represents the largest single-centre cohort of liner dissociation compared with the existing literature [3, 8, 13], however; it is not without limitations. The retrospective design of this study introduces inherent biases and limits our ability to fully control for all confounding factors. Therefore, it is challenging to draw definitive causal relationships between the observed factors and liner dissociation. Potential underestimation of this complication owing to loss to follow-up or database inaccuracies, especially with varying ICD coding, remains also a concern. Given our reliance on data from a single institution, it is possible that additional patients experienced liner dislocations but sought care at other facilities, leading to an underreporting of the true incidence. Furthermore, despite this single-centre study with standardized THA implantation procedures, individual surgeon variation may have been present. Limited access to detailed surgical reports and rotational CT analysis for all cases further constrains the study. To mitigate this, an algorithm designed to enhance the accuracy of anteversion and inclination calculations using pelvic plain AP radiographs was used, albeit recognizing the limitations in direct comparison with CT measurements due to methodological differences. Lastly, the exclusive use of 28 mm and 32 mm heads, as opposed to the 36 mm heads utilized in other centres for cups 52 mm and larger, may affect the generalizability of our findings and should be considered in the interpretation of the results.

## Conclusions

While liner dissociation is infrequent, it requires surgical revision in all affected cases.

Diagnosis is typically straightforward, as evidenced by the lateralization of the femoral head on X-rays and audible hip squeaking. Early diagnosis is fundamental to minimize potential damage to the acetabular cup, subsequently reducing the complexity of revision surgeries.

Key factors that contribute to the higher risk of liner dissociation include the use of conventional in comparison with highly crosslinked polyethylene. Appropriate acetabular cup anteversion may decrease the risk of liner disassociation, while using screws may increase the risk. It is important to interpret these findings with caution owing to the exclusive use of smaller femoral heads (28 and 32 mm) and a single implant design, which may limit their broader applicability. Moreover, the retrospective nature of this study introduces inherent biases, which must be considered when interpreting the results.

## Data Availability

All data generated or analysed during this study are included in this published article.
